# Opioid overdose prevention and naloxone rescue kits: what we know and what we don’t know

**DOI:** 10.1186/s13722-016-0068-3

**Published:** 2017-01-07

**Authors:** Todd Kerensky, Alexander Y. Walley

**Affiliations:** 1 Instructor of Medicine, Boston University School of Medicine, Boston Medical Center, 801 Massachusetts Avenue, Floor 2, Boston, MA 02118 USA; 2 Section of General Internal Medicine, Clinical Addiction Research and Education Unit, Boston University School of Medicine, Boston Medical Center, 801 Massachusetts Avenue, Floor 2, Boston, MA 02118 USA

**Keywords:** Naloxone rescue kits, Overdose prevention, Opioid overdose education

## Abstract

The opioid use and overdose crisis is persistent and dynamic. Opioid overdoses were initially driven in the 1990s and 2000s by the increasing availability and misuse of prescription opioids. More recently, opioid overdoses are increasing at alarming rates due to wider use of heroin, which in some places is mixed with fentanyl or fentanyl derivatives. Naloxone access for opioid overdose rescue is one of the US Department of Health and Human Services’ three priority areas for responding to the opioid crisis. This article summarizes the known benefits of naloxone access and details unanswered questions about overdose education and naloxone rescue kits. Hopefully future research will address these knowledge gaps, improve the effectiveness of opioid overdose education and naloxone distribution programs, and unlock the full promise of naloxone rescue kits.

## Background

As a leading cause of preventable injury and death, opioid overdose is a major contributor to worsening overall survival among middle-age white Americans and an increasing cause of mortality among all racial and age categories [1]. Increases in overdose have been driven by prescription opioids in the 1990s and 2000s and non-prescribed opioids in the 2000s and 2010s [2, 3]. In several communities, fentanyl has been recognized as a major contributor to increases in opioid overdose mortality since 2013 [2, 4, 5] and fentanyl derivatives such as acetyl fentanyl, furanyl fentanyl and carfentanil have been detected in drug seizures and overdose toxicology. The US Department of Health and Human Services has recognized opioid related overdose as a major public health concern and acknowledged three priority areas to address this crisis: opioid prescriber education, community naloxone access, and improved access to medications for opioid use disorder [6]. Each of these priority areas holds promise, though the strength of evidence for each of these is different. There is substantial, strong, and reproducible evidence in randomized clinical trials and well-designed observational studies that medications for treatment of opioid use disorder improve mortality, reduce opioid use, reduce infectious risks, reduce incarceration, and improves birth outcomes [7]. The effectiveness of opioid overdose education and community naloxone distribution (OEND) in reducing overdose deaths comes from a smaller research set which encompasses less rigorous study designs including: interrupted time-series analysis, pre-post studies, case series, and cross sectional studies [8]. There is less evidence that providing education on safe opioid prescribing will impact opioid overdoses and deaths.

As access to naloxone has improved, it is clear that much is known about community level overdose education and naloxone rescue distribution, but more research and knowledge is needed to optimize OEND as a valuable tool to combat the overdose crisis. This article summarizes what is known and highlights areas of knowledge gaps with respect to opioid overdose education and naloxone distribution.

## What we know

Naloxone is a potent opioid antagonist that is avid at the mu opioid receptor. It is FDA approved for emergency treatment of known or suspected opioid overdose with respiratory and/or central nervous system depression. Naloxone can be administered intravenously (IV), intramuscularly (IM), subcutaneously (SC), and intranasally (IN). Naloxone has no effect in people who are not taking opioids. OEND programs educate laypersons to recognize opioid overdose and instruct them how to administer naloxone to reverse respiratory depression. Access to OEND to potential overdose bystanders through community programs has expanded to 30 states since the late 1990s [9, 10]. Most OEND programs also provide education about overdose prevention by instructing potential rescuers to recognize known risk factors for overdose such as: mixing opioids with other sedatives, changes in drug potency or purity, using high doses of prescription opioids, and using opioids alone. Empowering people who use opioids to engage overdose prevention by recognizing and addressing modifiable risk factors is an important feature of OEND programs. A randomized controlled trial has shown motivational interviewing focused on overdose risk reduction to be superior to usual care in reducing self-reported opioid overdose risk behaviors in patients presenting to the emergency department with non-medical use of prescription opioids [11]. This is promising evidence that non-judgmental, goal directed interviewing can reduce risky behaviors in patients using opioids non-medically. Future studies will be needed to evaluate outcomes in other at risk groups for opioid overdose as well as whether including naloxone education and distribution may amplify reductions in risk taking behaviors.

Equipped with the education and training provided by OEND programs, naloxone can be administered by bystanders, whether that bystander is a person who also uses opioids, a friend, family member, acquaintance or first responder, such as police or firefighter personnel [12]. In some communities, people who use opioids and their social networks can obtain training and naloxone rescue kits at many different venues including needle-syringe access programs, inpatient and outpatient addiction treatment programs [9], primary care [13], and support group meetings [14]. It is important to recognize periods of abstinence resulting in loss of opioid tolerance, such as post-incarceration [15, 16] or after completing varying types of addiction treatment [17], as a high-risk time for an overdose event if relapse occurs. Therefore, providing OEND to opioid users and their social networks while they are engaged in addiction treatment, general health care, or the criminal justice system is critically important [18].

The legal framework in most states has shifted to promote access to naloxone kits by allowing health professionals to prescribe naloxone to third-party family members as well as making naloxone available without a prescription at retail pharmacies via a standing, often state-wide, prescription [19]. While community based naloxone programs remain the most common driver for naloxone distribution [9], naloxone prescriptions dispensed at US retail pharmacies have risen steeply since 2013 [20]. Retail pharmacy distribution is a promising way to improve access to naloxone, especially in rural areas which may be underserved by community OEND programs.

A mortality benefit from OEND is supported by observational evidence including an interrupted time series study that showed Massachusetts communities with OEND had reduced opioid overdose death rates compared to communities that did not have OEND [21] and a pre-post study in Scotland showed a reduction in overdose death rates among people released from prison [22]. A modeling study has demonstrated OEND to be cost effective for people who use heroin [23]. A trial published in 2016 found that co-prescribing naloxone rescue kits to patients treated with opioids for chronic pain in primary care resulted in reduced opioid-related emergency department visits [13]. This evidence in the context of an opioid overdose crisis is compelling for improving access to OEND; however, important questions remain unanswered.

## What we don’t know

### Who should receive opioid overdose education and naloxone rescue kits?

Existing evidence has focused primarily on training and delivering naloxone kits to people who use heroin (PWUH) via community based programs. This approach has proved fruitful, likely because PWUH are apt to use with others and/or be a bystander for another’s overdose. Thus, PWUH may be receptive to OEND intervention because they understand via their own experience the risks associated with opioid use and may be more likely to be present at an overdose event. It is not known if targeting OEND to friends, family members, acquaintances of opioid users, or the public could reduce mortality beyond the benefits of targeting OEND to PWUH.

Prescribers and pharmacists need guidance on who should receive naloxone rescue kits. One approach is to develop an overdose risk tool to help deliver OEND to people at risk for overdose. However, OEND should target people most likely to witness another’s overdose, in addition to focusing on individuals who are at risk themselves. Therefore, providing OEND to the social networks of those identified to be high risk for overdose might be especially efficient. Importantly, the social networks of people who use opioids may not be interacting with community based OEND programs or health care personnel may not be identifying them as potential beneficiaries of OEND.

### Should all patients receiving opioid therapy be offered naloxone co-prescribing?

Center for Disease Control and Prevention opioid prescribing guidelines released in 2016 recommend considering naloxone and overdose prevention education for patients and household members of patients prescribed opioids with a history of overdose, history of substance use disorder, higher opioid dosages (**≥**50 MME/day), or concurrent benzodiazepine use [24]. A study of co-prescribing naloxone as a universal precaution to patients on chronic opioid therapy for non-cancer related pain demonstrated reduced opioid-related emergency department visits after substantial, but not universal, uptake by prescribers and patients at a group of community health centers [13]. The patients who actually received naloxone rescue kits in this study were more likely to be those on higher doses of opioids and with previous opioid-related emergency department visits. How best to work with patients, their household members, prescribers, and pharmacies to get naloxone kits to a broader group of people exposed to opioids and at risk for overdose warrants further intervention development and implementation studies.

### Does OEND alter opioid prescribing practices?

The few studies that have examined how OEND may impact prescribing practices are mixed, and point to a nuanced relationship between offering naloxone to patients treated with prescription opioids. A qualitative study found that prescribers were conflicted about co-prescribing an opioid and an opioid antagonist [25]. Some providers interviewed for this study questioned whether opioids were contraindicated if naloxone co-prescribing was being considered. Whereas, some felt that the process of OEND alone might improve conversations about the risks of opioids. Providers felt that discussing and addressing overdose risk with OEND might reduce patients’ risk taking behaviors [25]. The previously mentioned study of co-prescribing as an intervention for patients on long-term opioids for chronic pain found reduced opioid-related emergency department visits, but prescribing naloxone had no net effect on the prescribed opioid dose [13]. A better understanding of how OEND and prescribing affect one another will require further study.

### How should the perception of risk compensation be addressed?

Some opioid prescribers [25] and policymakers [26] are concerned about “risk compensation,” meaning that having a naloxone rescue kit may increase risky opioid use. Well-designed observational studies have shown reductions in community level opioid overdose death rates where OEND has been implemented [21, 22], and reduced opioid-related emergency department visits among chronic pain patients who were co-prescribed naloxone rescue kits [13]. Thus, if there is any substantial increase in risky behavior due to risk compensation, it is outweighed by the important benefits of OEND. The concern about risk compensation is similar to other key public health interventions, such as seat belts to prevent motor vehicle deaths, vaccination and condoms to prevent sexually transmitted infections, and needle-syringe programs to prevent infectious disease transmission. Studies that have looked for risk compensation from naloxone access among people who use heroin, have found no clear evidence of it [27, 28]. This is likely because people who use opioids are very averse to naloxone induced opioid withdrawal, and opioid overdose education may reduce incremental risky behaviors. However, the perception of risk compensation is an important barrier to wider implementation of OEND. Therefore, implementation studies that address the perception of risk compensation among prescribers and minimize any increases in overdose risk behaviors resulting from OEND are warranted.

### How should naloxone be administered and at what dose?

#### Naloxone formulations and delivery systems

There are now four different formulations of naloxone that are used in naloxone rescue kits: (1) injectable naloxone that is drawn up out of a vial with a needle into a syringe with a dose concentration of 0.4 mg/1 ml, (2) an auto injector with audio prompts that administers a 0.4 mg intramuscular dose via a retractable needle, (3) a single-step nasal spray that administers a dose concentration of 4 mg/0.1 mL into one nostril, and (4) a multi-step nasal spray assembled by combining a pre-filled luer lock syringe with a nasal atomizer, that administers a dose concentration of 2 mg/2 ml, where 1 ml is administered to each nostril.^1^ OEND programs have favored syringe and vial intramuscular naloxone and multi-step nasal naloxone because of lower cost and early availability. Naloxone kits, regardless of formulation, generally include two doses, so that if the first dose does not result in spontaneous respirations, then a second dose may be administered.

Intranasal delivery has several potential benefits. First, no injection is required which facilitates layperson use because there is no fear nor risk of needle stick injury. Furthermore, the administration of a nasal spray requires less training than the administration of an injection, and the nose is typically readily accessible for administration. The new FDA-approved single-step nasal device does not require assembly providing a potential time saving advantage over the multi-step device. In 2016, information about how the single-step device is used in actual overdose events in people with opioid tolerance is lacking. Little is known about how active nasal naloxone is at opioid receptors in real world circumstances and how reproducible this method of delivery is.

The IM auto-injector also has benefits. The product was designed such that a layperson could easily and quickly administer naloxone via a one-piece device that instructs rescuers step-by-step, in real time through the process of delivering intramuscular naloxone. In addition to providing audible instruction, the device protects the needle via a plastic housing, thereby reducing unintended needle exposure. The cost of the auto-injector has been high and insurance coverage variable. Both cost and variable insurance coverage are important barriers to naloxone access. More information about real-life use of naloxone is needed to understand if this technology improves the correct and timely delivery of naloxone.

Some providers and health care systems may favor prescribing the single-step nasal device or the IM auto-injector because these devices are designed and FDA approved for bystander administration. OEND programs have favored the multi-step nasal device or intramuscular naloxone because the FDA approved devices are more expensive. It remains to be seen how the new, higher cost, FDA approved devices alter access to naloxone and potentially health outcomes.

#### Dosing

The most common and clinically important adverse effect of naloxone is precipitated opioid withdrawal. Ideally the dose of naloxone would be large enough to successfully reverse respiratory depression, yet small enough to avoid opioid withdrawal. It is not well known whether the dose of naloxone currently being utilized optimally balances the lifesaving properties of naloxone with risk of inducing withdrawal. The newer one-step nasal spray delivers naloxone at higher concentration and larger dose compared to intramuscular delivery of 0.4 mg/1 ml naloxone in healthy volunteers. It is expected, but not yet proven, that the one-step device will result in more successful reversals of respiratory depression compared to other delivery devices. However, it is also anticipated that naloxone induced withdrawal symptoms will be more frequent and possibly more severe because a higher dose of naloxone is used in this device. The trade-off between high doses of naloxone to prevent overdoses that fail to reverse with naloxone and the increased risk of withdrawal generally favors giving higher dose naloxone to help save lives. However, we do not yet know if naloxone induced withdrawal results in greater risk of repeat opioid use and recurrent overdose. In addition, it is reasonable to expect that locales with higher potency heroin and/or greater use of fentanyl or fentanyl derivatives would benefit more from higher dose naloxone. Thus, communities with more high potency opioid use may be more inclined to adopt higher dose naloxone kits, and accept the potential for more frequent and severe opioid withdrawal. More information about the frequency of opioid overdoses not responding to naloxone as well as a better understanding of what happens to patients after receiving naloxone, especially those who experience induced withdrawal, will help inform decisions regarding naloxone dosing.

### What happens after overdose rescue with naloxone to keep people safe?

Due to the neurobehavioral adaptations that occur among people who have an opioid use disorder, overdose survivors are unlikely to seek and engage wholeheartedly in addiction treatment immediately after receiving naloxone. Most survivors will be experiencing precipitated withdrawal from naloxone and will be having intense cravings to use opioids. Treating a survivor in withdrawal with a daily long-acting opioid agonist, like methadone or buprenorphine, is the most promising way to work towards engaging him or her in treatment and the best way to keep him or her safe from using more unsupervised opioids. Initiation of buprenorphine/naloxone in the emergency department (ED) has been evaluated in a randomized clinical trial of 329 opioid-dependent patients, of which 8.8% presented to the ED with an overdose event. This study demonstrated that initiation of buprenorphine/naloxone treatment in the emergency department with referral to further care resulted in improved engagement in medical care, less self-reported opioid use and less utilization of inpatient services at 30 days compared to referral to outpatient treatment or brief intervention [29]. This study is promising evidence, in a small number of patients at one academic medical center, that immediate initiation of agonist therapy may improve outcomes. This study did not report on outcomes in the subset of participants who required naloxone. Offering agonist therapy immediately after overdose requiring naloxone, whether the person experienced naloxone induced withdrawal or not, is an important area of future research to improve care after an overdose event.

Some have called for mandated treatment after an overdose, which has unclear efficacy as well as ethical and civil rights implications [30]. Other potential interventions include survivor-centered harm reduction and treatment outreach to people who have overdosed and their social networks. The Anchor Recovery Coach Program in Rhode Island provides on-call recovery coaches to survivors presenting to the emergency department [31]. When the survivor has a disease that involves using opioids despite adverse consequences, engaging others in the survivor’s social network to promote overdose prevention strategies and support seeking treatment may reduce the likelihood of the next overdose. Programs providing care, counseling, and OEND immediately after an overdose to survivors and their social networks may provide a unique opportunity to reduce repeat opioid overdose and encourage engagement in further care, and therefore need to be evaluated.

### What are the subacute effects of non-fatal opioid overdose and how can they be treated or prevented?

Complications from non-fatal overdose include but are not limited to: aspiration, anoxic brain injury, nerve palsies, and trauma related injuries. The incidence of these complications has not been well described. Additionally, it is not known how long and in what setting people should be monitored after naloxone rescue to reduce the risks of overdose complications.

### What kind of training should be provided with naloxone distribution?

In many communities, laypersons can obtain naloxone at different locations which provide varied information about overdose and naloxone. This education differs across several potentially important variables which could alter the impact of OEND programs. First, the material may be conveyed in multiple formats. Commonly utilized techniques include: didactic education, hand-out media such as pamphlets, and/or media like video that is consumed while at the training session. Some programs utilize hands-on training via a demo device, while in some locations this may not be available. Second, who delivers the content and for how long is not standardized. In some places, trained community members provide instruction while in more formal medical settings, physicians, nurses, or pharmacists may lead overdose education and naloxone training. Third, the length of OEND training also fluctuates between settings, trainers, and trainees. Space, trainer time, and trainee time may be limited and trainee knowledge and cognition may require different training intensity. Further study into how best to educate laypersons about overdose and naloxone is needed to optimize the effectiveness and efficiency of OEND. Standardization of the core elements of OEND training, yet allowing for some flexibility of other elements of the training, will help ensure the future rescuer achieves a minimum competency and comfort level with naloxone. Standardizing and tailoring OEND curriculum will need to evolve as more naloxone devices with various concentrations, doses, and delivery mechanisms become available to wider groups of potential rescuers.

The American Heart Association (AHA), in its most recent update in November 2015, incorporated naloxone into its emergency response algorithms [32]. This presents a new opportunity for opioid overdose education and naloxone training to occur along with AHA emergency response trainings. While OEND can be incorporated into AHA trainings, it is not known if more advanced resuscitation skills, such as chest compressions and automatic defibrillator use should be provided at existing OEND programs. These additional skills may be useful in opioid overdose rescues, however teaching these skills may result in additional barriers to naloxone access in the community. AHA cardiopulmonary resuscitation course includes proficiency testing prior to certification and requires a refresher course every 2 years to maintain certification. It is not known whether testing proficiency or requiring recertification might improve overdose related clinical outcomes.

### After recognizing an overdose, what is the correct order of actions?

OEND programs and the AHA instruct rescuers to call for help, start ventilation or CPR, deliver naloxone, remain with the person until help arrives, and place victim in the rescue position. OEND programs, naloxone package inserts, the World Health Organization and the AHA have various recommendations regarding the specific order in which these key steps should be performed. It is not known if or how the order of these rescue steps could alter clinical outcomes. Further study will hopefully standardize how we respond to opioid overdose events.

### How do local and state laws affect OEND?

The legal framework in which OEND programs operate may have significant implications for how effectively naloxone reduces overdose deaths. OEND programs exist in different state and local legal environments. Many states have attempted to limit civil or criminal liabilities for responding to an overdose, as well as administering, prescribing or distributing naloxone. These laws differ by location but generally encourage wider access to OEND and help encourage rescuers to call for emergency services by reducing fears of legal repercussions when first-responders, including police, arrive. Currently, we do not know how OEND effects a rescuer’s decision to call for emergency help. OEND programs recommend calling for emergency services, but rescues are occurring without summoning professional help. This may be due to fear of legal consequences to either the overdose survivor or the rescuer, but other less well known factors may also discourage calling for help. The implications of layperson rescue without the survivor interacting with professional help after this event are not known. It would be informative to compare effectiveness and distribution of naloxone in areas that have different legal protections and evaluate how OEND effects professional help seeking after overdose to understand and address potential barriers to naloxone use.

### Would dedicated CME training programs for prescribers improve naloxone prescribing and distribution?

Most clinicians experience with naloxone occurs in a hospital or pre-hospital setting where the antidote is delivered by nurses or first responders. Relatively little is known about prescribers’ knowledge about naloxone for community use by lay bystanders. Improving prescriber knowledge about community naloxone programs and prescribing naloxone via pharmacies may help distribute naloxone more broadly, especially in communities that do not have access to an OEND program.

### What are validated research and clinical measures of overdose and overdose risk behaviors?

We have called for additional research to improve the clinical and health system benefits of OEND. High quality research relies on well-defined clinical events and outcomes as well as validated measures. Fatal opioid overdoses are defined typically via cause of death fields on death certificates, which are available in all communities. However, standardization of what constitutes a fatal overdose event is needed to help facilitate research across jurisdictions and improve epidemiologic reporting. Additionally, there are not validated definitions of non-fatal opioid overdose, whether measured by self-report, clinical, or administrative data. For opioid overdose, the basic components are exposure to an opioid that results in unresponsiveness and respiratory depression. Definitions of overdose which require that help was sought or that naloxone was administered likely increase specificity, but sacrifice sensitivity. Opioid overdose rescues may occur without calling for help and/or administering naloxone. If the definition of non-fatal overdoses requires these elements, it is likely that some non-fatal opioid overdoses would not be classified appropriately, therefore reducing sensitivity. On the other hand, a firm definition of non-fatal opioid overdose will be needed to exclude other causes of unresponsiveness and/or respiratory depression to improve specificity. Universally accepted definitions of both fatal and non-fatal opioid overdose events will be needed to help improve OEND research in the future.

Overdose risk behaviors have been well described. Assessing overdose risk and educating patients about it are key elements of overdose prevention. We have yet to develop a validated, widely applicable overdose risk measure that is useful clinically or for research.

## Conclusion

The opioid use and overdose crisis is persistent and dynamic, garnering much attention from the public, policymakers and public health officials. In an effort to curb opioid related overdoses and deaths the federal government has prioritized increasing prescriber education, improving access to treatments for opioid use disorder and naloxone. As an antidote to opioid overdoses, naloxone has proven to be a valuable tool in combating overdose deaths and associated morbidity. Further investigation into important knowledge gaps will help unlock the potential of naloxone needed to address the pervasive opioid overdose crisis.

## Abbreviations

OEND: opioid overdose education and naloxone distribution
PWUH: people who use heroin
BLS: basic life support
ACLS: advanced cardiac life support
AHA: American Heart Association
IV: intravenous
IM: intramuscular
SC: subcutaneous
IN: intranasal
ED: Emergency Department

## Footnotes

1 Nasal naloxone delivered via syringe and nasal atomizer as 2 mg/2 ml is not FDA approved.
